# Diplopia due to a neurovascular compression

**DOI:** 10.22336/rjo.2022.15

**Published:** 2022

**Authors:** Dana Margareta Cornelia Dăscălescu, Vasile Potop, Valeria Coviltir, Maria Cristina Corbu, Cristina Dijmărescu

**Affiliations:** * Clinical Ophthalmology Emergency Hospital, Bucharest, Romania,; ** “Carol Davila” University of Medicine and Pharmacy Bucharest, Romania,; *** Clinical Emergency Hospital Bucharest, Romania

**Keywords:** horizontal diplopia, neurovascular compression, sixth nerve palsy

## Abstract

A 36-year-old female patient presented to our clinic with a two months history of diplopia and dizziness. The symptoms appeared gradually and increased in frequency and intensity. She had no significant medical history and she did not take any medication.

A full ophthalmological consult was performed, which revealed restricted ocular motility in the left eye (LE), in left gaze. Otherwise, the examination showed no pathological findings: best corrected visual acuity (BCVA) both eyes (OU) 1 (Snellen chart), normal slit lamp examination and pupillary reflexes, normal intraocular pressure (IOP) and fundus aspect.

Diplopia tests revealed a horizontal diplopia, exacerbated in left gaze. Sixth nerve palsy suspicion was raised and the patient was directed to the neurology department. Following magnetic resonance imaging, with angiographic sequence, a complex intracerebral vascular malformation that interacted with the cranial nerves and determined horizontal diplopia, was found.

For a correct diagnosis, we needed a good collaboration between various medical specialties, especially ophthalmology and neurology, because patients with diplopia often present for the first time at the ophthalmologist.

**Abbreviations:** BCVA = best corrected visual acuity, IOP = intraocular pressure, LE = left eye, RE = right eye

## Introduction

Binocular diplopia patients usually present for the first time to the ophthalmology department. After a complete checkup, in cases of a cranial nerve palsy suspicion, they are directed to the neurology department for further examination. 

Horizontal binocular diplopia is often determined by a sixth cranial nerve palsy. Causes can be various and include infarction, aneurysm, cavernous-carotid fistula, primary tumors, neoplastic infiltration, demyelination, infections, meningitis, idiopathic inflammation, high or low intracranial pressure, etc. [**1**,**2**]. The nerve can be affected on any portion of its trajectory: brainstem fascicle, in the subarachnoid space, in the petrous apex, in the cavernous sinus or in its orbital apex [**3**]. 

## Materials and methods

We present the case of a 36-year-old female patient who came to our clinic with a two months history of horizontal diplopia, headache, and dizziness. The symptoms appeared gradually and increased in frequency and intensity. The patient had no significant medical history and did not take any medication. 

She was submitted to a complete ophthalmologic examination that included refraction, visual acuity assessment, slit lamp examination, intraocular pressure measurement (IOP), fundus examination, optic nerve assessment and visual field test. Best corrected visual acuity was 1 in both eyes (OU), with a correction of +0.50 Dsph, using a Snellen chart. IOP Goldmann OU was 15 mmHg and slit lamp examination did not reveal any pathological findings, direct and consensual pupillary reflexes being intact. 

Ocular motility revealed no deviation on midline, while on horizontal eye movement it showed an abduction deficit of the left eye (LE), with intact right eye (RE) movements. We performed a red glass diplopia test that objectified horizontal diplopia with a LE abduction deficit. 

**Fig. 1:**
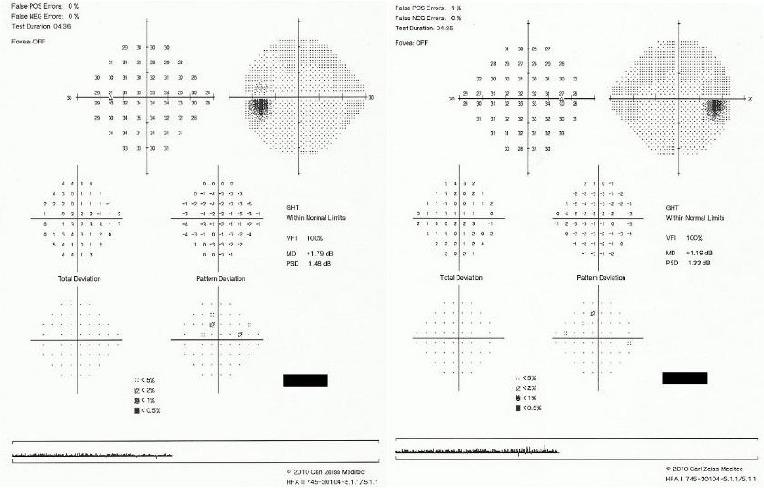
Visual field examination

Visual field assessment was performed before the dilated fundus examination and was within normal limits (**Fig. 1**). 

Fundus examination was then performed and revealed normal optic discs, with a cup/ disc ratio of 0.3, normal vascularization, attached retina and no pathological findings. Optical coherence tomography also revealed normal peripapillary retinal nerve fiber layers and retinal ganglion cells in both eyes.

## Results

We established the diagnosis of sixth nerve palsy in the LE and directed the patient to the neurology department. 

The neurological examination revealed that the patient was oriented to time and place, had no notable motor, coordination or sensory deficits, intact language function, preserved deep-tendon reflexes, down-going plantar reflex bilaterally, cranial nerve exam: no ocular deviation on midline, intact direct and consensual pupillary reflexes, on horizontal eye movement - abduction deficit of the LE - left abducens nerve palsy (with binocular horizontal diplopia reported by the patient), otherwise intact cranial nerve function.

**Fig. 2:**
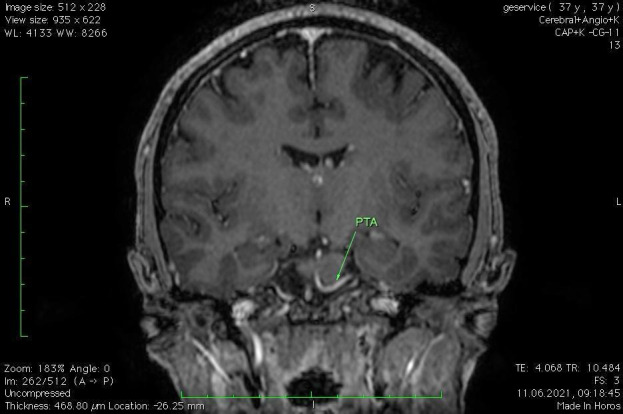
Persistent trigeminal artery (PTA)

**Fig. 3:**
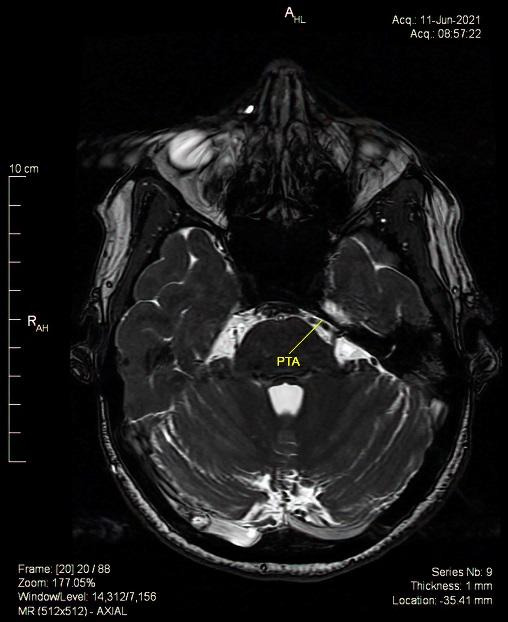
Persistent trigeminal artery (PTA) in contact with abducens and trigeminal nerve

**Fig. 4:**
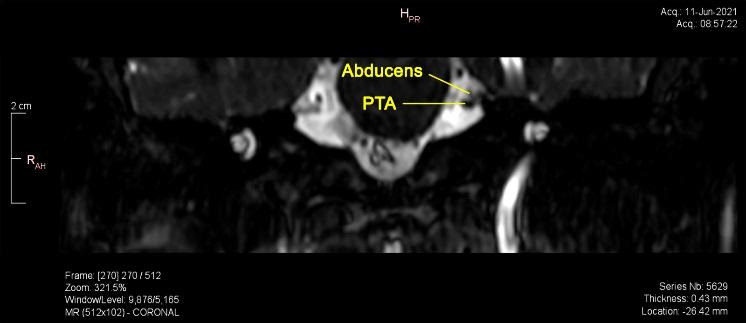
Persistent trigeminal artery (PTA) in contact with abducens nerve

Brain magnetic resonance imaging (MRI) revealed multiple bilateral vascular and degenerative lesions, unspecific, subcortical (vascular microangiopathy). The tortuous artery that surrounds the cisternal trajectory of the left trigeminal and abducens nerves associated vascular-nervous conflict. Moreover, the MRI revealed a complex anatomic variant within the vertebral-basilar system, carotid artery connections, persistent trigeminal artery (PTA) variant and fetal type posterior cerebral artery (**Fig. 2**-**4**). 

The neurological consult and patient symptomatology ruled out infectious or inflammatory disease, cavernous-carotid fistula, demyelination, meningitis, idiopathic inflammation and high or low intracranial pressure, the ophthalmological consult ruling out the ocular pathology. The MRI excluded demyelination, infarction or aneurysm, tumors or neoplastic infiltration and revealed the neurovascular compression due to a persistent trigeminal artery variant.

The patient was referred to the neurosurgery department, but this anatomic variant could not be approached surgically due to its location. For this reason, in order to help the patient improve her quality of life, we prescribed prism glasses and the evolution was positive, the patient adapting well with the glasses.

## Discussion

The abducens nerve can be compressed due to a vascular anomaly in the same way that other nerves can be, but due to its long trajectory, clinical symptoms can appear late in the course of the disease [**4**-**6**]. Other diplopia causes such as mass lesions that protrude into the cerebellopontine cistern, infectious or inflammatory disease (usually affecting the cisternal segment) can be easier to identify due to their systemic implications and symptoms [**7**,**8**]. 

A persistent primitive artery is a rare cause of neurological deficits. The primitive trigeminal artery is the most common carotid-vertebrobasilar anastomosis, and can be found in 0.1-1% of the population [**9**]. Anomalies involving this persistent artery may lead to other visual disturbances as well, such as visual field defects [**10**].

## Conclusions

Patients with visual symptoms, even transitory, usually present to the ophthalmology department, often ignoring other symptoms such as headache or nausea. For this reason, ophthalmologists need to make a clear separation between an ophthalmologic cause and a neurological one and make the patient understand the importance of an early presentation and diagnosis in order to have the best therapeutic options available. 

The relation between the ophthalmic examination and the neurological examination is very important for a proper diagnosis, for the follow up, treatment and adverse effects.

**Conflict of Interest statement**

The authors state no conflict of interest.

**Informed Consent and Human and Animal Rights statement**

Informed consent has been obtained from all individuals included in this study.

**Authorization for the use of human subjects**

Ethical approval: The research related to human use complies with all the relevant national regulations, institutional policies, and is in accordance with the tenets of the Helsinki Declaration, and has been approved by the review board of Clinical Ophthalmology Emergency Hospital, Bucharest, Romania.

**Acknowledgements**

None.

**Sources of Funding**

None.

**Disclosures**

None.

**Contribution**

All authors had equal contribution to this paper, equal with the first author.
